# Phantom decoys alter perceived missed opportunity costs and increase risky foraging decisions

**DOI:** 10.1093/beheco/arag078

**Published:** 2026-07-11

**Authors:** Finn C G Parker, Catherine J Price, Jenna P Bytheway, Clare McArthur, Peter B Banks

**Affiliations:** School of Life and Environmental Sciences, Science Road Cottage A10, Science Road, Camperdown, The University of Sydney, Sydney, NSW 2050, Australia; School of Life and Environmental Sciences, Science Road Cottage A10, Science Road, Camperdown, The University of Sydney, Sydney, NSW 2050, Australia; School of Life and Environmental Sciences, Science Road Cottage A10, Science Road, Camperdown, The University of Sydney, Sydney, NSW 2050, Australia; School of Life and Environmental Sciences, Science Road Cottage A10, Science Road, Camperdown, The University of Sydney, Sydney, NSW 2050, Australia; School of Life and Environmental Sciences, Science Road Cottage A10, Science Road, Camperdown, The University of Sydney, Sydney, NSW 2050, Australia

**Keywords:** context-dependent decision-making, risk-sensitive foraging, ecological rationality, pest management, information use, missed opportunity cost

## Abstract

Foraging decisions about risk and reward are driven by an option's perceived value relative to foregone alternatives, ie missed opportunity costs (MOCs). Information that alters perceptions about alternatives (ie perceived MOCs) should therefore influence animal behavior, yet perceived MOCs are overlooked in wildlife management and rarely manipulated. Phantom decoys are attractive but inaccessible options (ie missed opportunities) that change decision-making, seemingly by altering perceptions of MOCs. Using phantom decoys, we conducted 2 experiments to: (a) test if misinformation about MOCs alters risk versus reward foraging decisions; and (b) assess whether decoys can increase trapping rates by changing how animals trade off trap danger for rewards inside. Firstly, we conducted a giving-up density (GUD) experiment on wild house mice (*Mus musculus*) in large natural enclosures, creating patches comprising safe and risky trays of high- and low-quality foods, with decoys at half the patches. Where decoys were present, mice foraged longer in risky trays, accepting greater risk at these patches compared with otherwise identical patches without decoys. Secondly, in a field trapping experiment, we positioned decoys beside trap entrances, and compared capture rates to controls. More mice were captured in traps with decoys outside them. Our results demonstrate how information alone can shape perceptions of MOCs to increase risky behavior. Behavioral responses to “irrelevant” alternatives (ie phantom decoys) have traditionally been considered irrational. Our findings suggest that attractive resources provide valuable information about the environment, even if inaccessible, highlighting the potential for decoy information to alter decision-making to improve wildlife management outcomes.

## Teaser

Like us, animals do not like missing out, and tempting, out-of-reach options can make them take more risks to pursue what is available. We show that attractive but inaccessible food items (phantom decoys) cause mice to invest more effort while accepting greater risk, because they perceive alternatives elsewhere as worse. These findings show how animals consider opportunity costs, with implications for improving traps, lures, and other wildlife management tools.

## Introduction

Animals in search of food must constantly select amongst alternative foraging opportunities, often trading off the quantity and quality of food they can obtain (eg Wright et al. 1998), the physical cost of obtaining it (eg Weimerskirch et al. 2003), and the risk incurred by foraging for it (Lima and Bednekoff 1999). Importantly, there is no absolute value, cost or risk to any 1 opportunity, as each is relative to the value, cost and risk of other opportunities an animal perceives to be available (Stephens 2008). Opportunities animals forego when they make a decision represent the missed opportunity cost (MOC) of that decision, and changes in variables such as marginal value and risk are ultimately changes in MOCs because they only matter relative to available alternatives. For example, foraging in 1 patch when better options are available incurs an MOC in the form of time and energy that could have been more efficiently spent elsewhere (Olsson and Molokwu 2007). Animals that incur the lowest MOCs while foraging can allocate the most time and energy to enhancing their fitness (Winterhalder 1983), so animal perceptions of MOCs should play a fundamental role in the decisions they make. Crucially, because animals must estimate the value of alternatives based on the information available to them, MOCs are determined by perceived rather than actual opportunities. However, how animals perceive and evaluate information about MOCs remains poorly understood.

Minimizing MOCs requires information about what alternatives are available in the environment to reduce uncertainty and make more informed decisions (Inglis et al. 2001). The information an animal has about its environment when it makes a decision forms its “prior” information state (Valone 1991), and includes information about the distribution and quality of different resources. In complex, heterogenous environments, resources vary spatially and temporally (Stephens and Krebs 1986), meaning animals aiming to maximize foraging efficiency must continuously collect new information to better exploit environmental resources (Olsson and Brown 2006; Valone 2006), monitor changes in patch safety (Lönnstedt et al. 2012), and track the reliability of food cues (eg Price and Banks 2012; Parker et al. 2023b). Animals that continually sample available resources and refine their information about opportunities in the environment can make better decisions that reduce their risk of incurring high MOCs (Olsson and Brown 2006). In this sense, information determines the alternatives animals perceive to be available, and therefore the MOCs associated with their decisions. Animals forage in environments full of information about opportunities that are not necessarily accessible to them (ie MOCs), yet few studies have examined how manipulating this information directly can alter foraging decisions.

Because animals are so dependent on information to make decisions, new wildlife management approaches have emerged that use sensory information to change animal behavior and decision-making (Blumstein and Berger-Tal 2015; Elmer et al. 2021). Animals have evolved adaptive behavioral responses to sensory information that are well suited to the environments they evolved in (ie, they are ecologically rational) (Todd and Gigerenzer 2007). However, such adaptive responses to environmental cues can become maladaptive (ecologically irrational) when information does not accurately represent the environment (eg ecological and evolutionary traps; Schlaepfer et al. 2002). This is the basis for sensory misinformation in wildlife management, as predictable, adaptive responses to information by animals are exploited by deploying information that does not reflect the true environment (Price et al. 2022). If animals rely on information about MOCs to make foraging decisions, then misinformation that manipulates perceptions of MOCs could provide new opportunities for wildlife managers to alter animal decision-making.

Phantom decoys can act as a type of misinformation, and are well known to alter decision-making in humans (Pettibone and Wedell 2000) and a variety of animals, including cats (Scarpi 2011), fish (Maia and Volpato 2019), and bees (Tan et al. 2015). They are attractive but unavailable options in a decision (ie similar to a missed opportunity) that alter preferences for available alternatives (Pratkanis and Farquhar 1992). Behavioral responses to phantom decoys have been used to understand seemingly “irrational” decision-making, because they violate independence of irrelevant alternatives, a key principle of economically rational choice. For example, it has been suggested that the addition of a third, unavailable option should not increase the likelihood of choosing either of 2 available options (Rieskamp et al. 2006), or alter their relative preference (Ray 1973). However, from an ecological perspective, every foraging opportunity must be weighed up against the background of alternatives that will potentially be missed out on (Stephens 2008). Thus, despite being inaccessible, a phantom decoy may still become part of that background by providing information about an attractive but unavailable option. In doing so, they expand the set of alternatives that an animal has information about, which can alter the perceived benefits of exploiting or avoiding a particular option. That is, phantom decoys should alter how animals evaluate the tradeoff between exploiting 1 option and leaving to pursue other opportunities. As a result, phantom decoys may change perceived MOCs despite having no effect on the actual distribution of resources.

While phantom decoys are likely to alter perceived MOCs, it is unclear what the direction of the effect on the forager will be. An attractive but unavailable option in a foraging patch might signal that better foraging opportunities exist elsewhere, raising perceived MOCs at patches containing a decoy. Alternatively, it might signal to the forager that options at the patch containing the decoy are superior relative to alternatives, lowering perceived MOCs. In either case, because MOC contributes to the marginal value of energy gained from a patch (Brown 1988), a change in MOC should not appear as an uniform shift in foraging effort, but as a change in how readily animals accept the costs of exploiting that patch. The marginal value theorem predicts that when perceived MOCs are low, each food item is worth more, so animals should accept greater predation risk and lower-quality food. When perceived MOCs are high, animals should forage more selectively, favoring safer and higher-quality opportunities (Charnov 1976; Brown 1988). Regardless of the effect's direction, if decoys do alter perceived MOCs, they have the potential to change animal decision-making, including in a wildlife management context.

The potential to use MOCs to change decision-making in wild animals has rarely been explored (but see Orlando et al. 2023; Jarvis et al. 2026), despite MOCs underpinning the outcomes of many wildlife interventions. Wildlife management and conservation efforts are frequently hampered by low interaction rates with baits and traps, likely because animals perceive their foraging opportunities to be better (or less risky) elsewhere (Krebs et al. 1994; Garvey et al. 2020). The probability of an animal interacting with a management stimulus (eg a trap, bait, or protected asset) is determined by the value of the stimulus (eg the attractant), the perceived risk of interacting, and the perceived MOCs of choosing to interact (Garvey et al. 2020). Traps and baits are often perceived as risky, due to factors such as neophobia (Barnett 1958, 1988), and fear of confinement (Hernández et al. 2018), particularly if animals have been trapped before (Camacho et al. 2017). An attractive lure may not be sufficient to compensate for this risk, especially if MOCs are high (eg they have many alternative foraging opportunities). However, if decoy information about an attractive but inaccessible opportunity decreases the perceived MOCs of interacting with a management stimulus, it may increase the probability of an interaction (ie trap entry). On the other hand, if an attractive but inaccessible opportunity beside a management stimulus increases the perceived MOCs of interacting, decoys may lower the probability that animals will interact.

Here, we report on a foraging experiment and a field trapping experiment on wild house mice to test if and how misinformation about MOCs in the form of phantom decoys can alter the perceived MOCs of foraging in a patch and interacting with a management stimulus, respectively. For our foraging experiment, we used the giving-up density (GUD) framework (Brown 1988) in large purpose-built mouse-proof enclosures. Giving-up densities (GUDs) are the number of food items left in a foraging patch after a set amount of time (generally overnight). They reflect patch-quitting decisions representing the instantaneous harvest rate when the forager stopped foraging (H), which is predicted to be driven by a combination of energetic cost (C), predation risk (P), and missed opportunity costs (MOC) (H=C+P+MOC) (Brown 1988). We provided foraging patches for mice, manipulating predation risk (risky and safe treatments) using ground cover (Powell and Banks 2004) and energetic cost using food quality (high and low-quality), and tested how foraging decisions changed in response to decoys. If decoys alter perceptions about alternatives and therefore perceived MOCs, we predicted that responses to predation risk and/or food quality would differ between decoy and nondecoy patches. For our trapping experiment, we conducted 2 trapping sessions over 2 yr, deploying 270 traps for a total of 1,530 trap nights. We positioned highly attractive phantom decoys directly beside trap entrances and compared capture rates and probability to control traps containing the same bait but no decoy, and procedural control traps with the same bait and a decoy inside the trap. If decoys decreased MOCs associated with interacting with traps, then we expected higher capture success in traps with decoys outside.

## Materials and methods

### Foraging experiment

#### Study location

**Figure 1:**
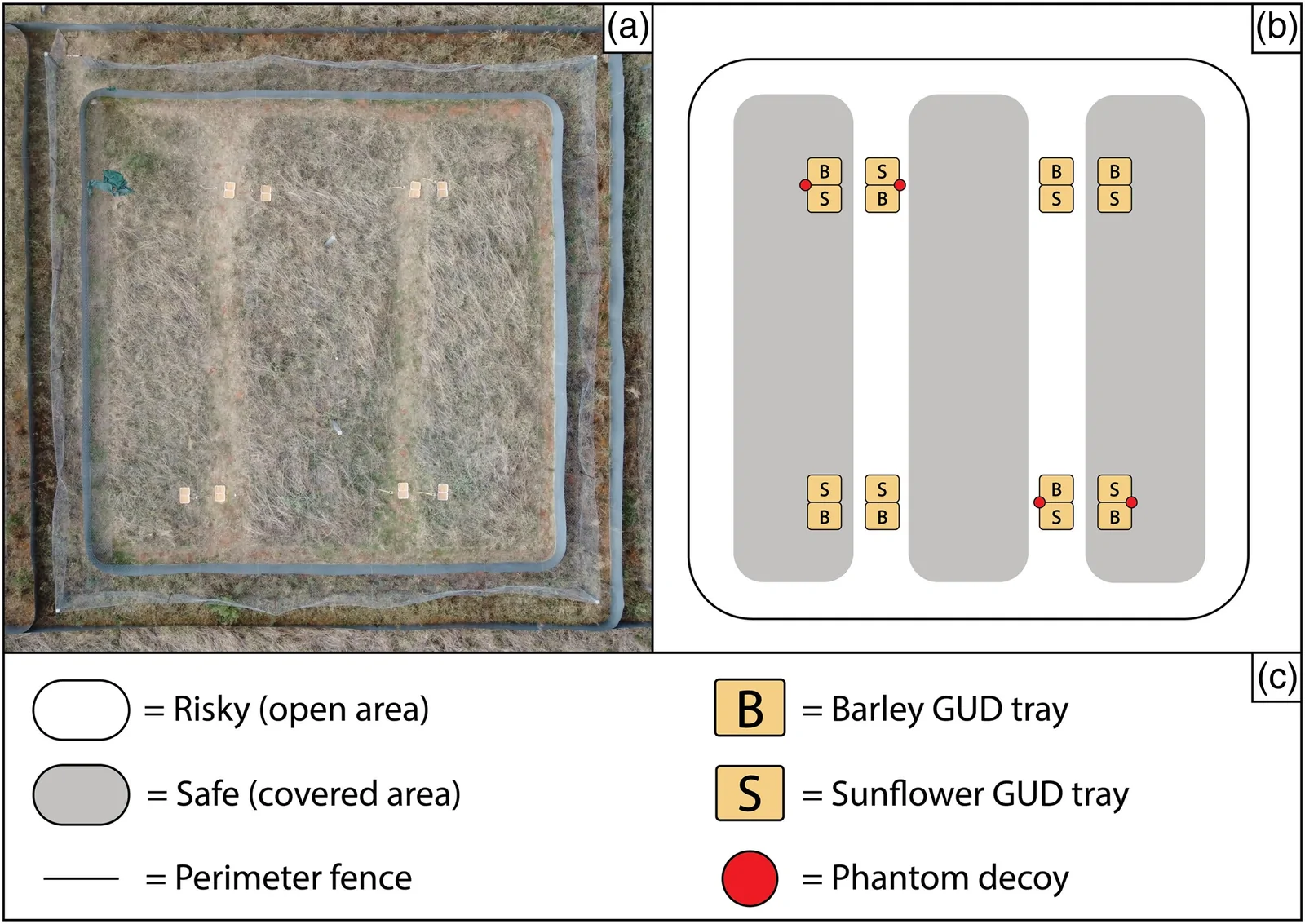
a) Aerial view of enclosure setup for foraging experiment. GUD trays were arranged in patches of 4 in each of the 4 quarters of the enclosure. Each patch contained 2 pairs of trays, 1 safe (in vegetation cover) and 1 risky (out in open). Each pair contained 1 tray with 20 sunflower kernels (high-quality food) and another with 20 barley seeds (low-quality food). Two patches at opposite corners contained phantom decoys positioned directly beside both pairs of trays; b) shows example schematic for clarity (not to scale); c) legend for panel b).

This study was conducted in 9 large outdoor enclosures (Figure S1) in Walpeup, Victoria, Australia (−35.138336, 142.023412) in April 2023, using wild house mice (*Mus musculus*). Enclosures comprised a perimeter fence measuring 15 × 15 m with an additional outer fence providing a 1 m wide buffer zone around the perimeter of each enclosure (Fig. 1). The total area that mice were able to forage in was 225 m^2^, which is larger than the average home range of a mouse during breeding season (Krebs et al. 1994). However, previous studies conducted in the same enclosures have shown that 1 mouse can visit up to 75/100 individual patches in a single night (Carthey et al. 2011; Price and Banks 2017), demonstrating that the entire enclosure is discoverable. Enclosures were mouse- and predator-proof, with 2 galvanized steel fences both approximately 1.5 m high and dug 50 cm underground to prevent mice climbing or digging their way out. Avian, reptilian and mammalian predators were prevented entry by chicken wire fencing (5 cm aperture, 2.5 m high) surrounding the entire enclosure (for additional details, see Barker et al. 1991).

The enclosures contained vegetation typical of the surrounding area, which allowed manipulation of ground cover. Vegetation within 0.75 to 1 m of interior perimeters was removed to discourage mice from inhabiting the edges of enclosures and to prevent escape. Two strips approximately 1 m wide and 4 m from the edge of the enclosure and 4 m apart were mowed the length of the enclosure to create open patches with no vegetation cover (Fig. 1).

#### Mouse trapping

We found evidence that mice were already present in enclosures (ie burrows and diggings), likely due to incomplete removal after a previous experiment, so we set 20 traps in each enclosure to estimate existing mouse numbers. Traps were baited with wheat seeds, because wheat would not be used in our experiment, but is still a highly desirable food for mice (Parker et al. 2023a). Trapped mice were given unique shave marks before being released back into the enclosures to provide a minimum number known alive (MNKA) as our estimate of mouse density (given the low recapture rates typical of house mice that prevents traditional mark-recapture estimation methods, Krebs et al. 1994). Individuals were removed to maintain an MNKA of approximately 10 to 12, and to maintain an even sex ratio, minimizing potential for confounding social and sex-related factors (eg Palanza et al. 2001).

#### Experimental set up

We used the GUD framework (Brown 1988) to test mouse foraging responses to phantom decoys at patches. Because patch-quitting decisions are driven by energetic cost, predation risk and MOCs, controlling food value and risk allows us to explore through GUDs how decoy presence changes perceived MOC. We had 4 foraging “patches” per enclosure, each consisting of 4 foraging trays located in each quarter of the enclosure, at the end of the mowed vegetation strips, approximately 3 m from the edge (Fig. 1). Trays were foil BBQ trays (32.5 × 26.3 × 6.7 cm) and contained 2.5 L of sand collected and sifted from a nearby paddock. The 4 trays in each patch were arranged into pairs, 1 pair assigned as risky and the other as safe, with each pair containing a high-quality food tray and a low-quality food tray. Safety and risk were simulated with ground cover, which is known to influence mouse perceptions of safety (Powell and Banks 2004). Safe tray pairs were located in vegetation cover (<50 cm from open strips), and risky tray pairs were located in open strips (<0 cm from cover). To test for food quality effects, a tray in each pair contained either 20 sunflower kernels (a preferred food of mice), or 20 barley seeds (a less-preferred food) (Price and Banks 2017). Decoys were present at 2 patches on opposite corners per enclosure. Phantom decoys were universal small mammal bait mix (peanut butter, rolled oats, honey)—an extremely attractive food for mice (eg peanut butter, Witmer et al. 2014; and peanuts, Price and Banks 2017)—rolled into bait balls (∼28 g) and contained in cappuccino shakers (small stainless-steel containers with mesh lids) so that mice could smell the bait but not access it. Decoys were attached to wooden stakes with duct tape, and stakes were hammered into the ground. Both pairs of trays at decoy patches had phantom decoys positioned immediately beside them (4 decoys per enclosure, Fig. 1).

We predicted that decoys would influence foraging at patches where they were positioned, but it was also possible that decoys might alter foraging decisions across the entire enclosure. To control for this, 4 of the 9 enclosures were initially allocated as decoy enclosures and the remaining 5 as control enclosures. Control enclosures were set up identically to decoy enclosures, except with no decoys present. All enclosures were run as both control enclosures and decoy enclosures, with treatments reversed in between. Control enclosures were used to provide a baseline against which to compare responses to decoys, and to determine if decoys were influencing mouse foraging across an entire enclosure, rather than only at decoy patches.

GUDs were measured as the number of sunflower and barley seeds remaining in trays each morning, with lower quantities of food remaining in a tray equating to higher foraging effort by mice at the tray (Brown 1988). Before the experiment began, all enclosures were set up without decoys to allow mice to acclimatize to foraging on trays. This process occurred while trapping was still taking place, which allowed us to observe how GUDs stabilized as we removed mice from enclosures. Once all enclosures had stable GUDs (at an MNKA of 10 to 12), traps were removed, and we allowed 2 more nights for acclimatization. Once decoys were added to half of the enclosures, GUDs were counted in all enclosures each morning for 2 nights before treatments were swapped and GUDs counted for 2 more nights.

#### Statistical analysis

To test for decoy effects within enclosures, we used a generalized linear mixed model with a beta-binomial distribution and logit link function in R version 4.4.1 (R Core Team 2024) using the glmmTMB package (Brooks et al. 2017). Our model tested whether GUD (our dependent variable) varied according to decoy presence, risk, food type, and night, including interactions between decoy presence, food quality and risk. We included enclosure as a random effect to account for variation amongst enclosures, with nested random effects for patch and tray, where patch accounted for spatial clustering of trays within enclosures and tray accounted for repeated measures across nights.

To compare decoy effects with baseline foraging behavior (ie foraging in enclosures with no decoys), we also analysed the full dataset including control enclosures. A beta-binomial GLMM was fitted with patch type (control, nondecoy patch, decoy patch), risk, food quality, and their interactions as fixed effects, with enclosure, patch, and tray included as nested random effects.

Model fits were compared, and the final model selected using Akaike's Information Criterion (AIC) (Akaike 1998). A beta-binomial distribution was chosen as GUD data were over dispersed, positively skewed, and bounded (ie between 0 and 20 as the maximum number of food items that could be found). Model validation for these models and all generalized linear mixed models was conducted using the “DHARMa” package (Hartig 2022) to ensure no significant issues with dispersion, outliers, or model specification.

To interpret treatment effects, we used estimated marginal means derived from the fitted models using the “emmeans” package in R (Lenth 2024). Pairwise comparisons among relevant treatment combinations were conducted with Tukey adjustment to control for multiple comparisons. These comparisons were used to examine differences between risky and safe patches within each decoy condition, differences between food types within each decoy condition, and differences among patch types within levels of risk and food quality. Adjusted *P*-values were interpreted alongside estimated effect sizes and confidence intervals, rather than against a fixed significance threshold.

Because GUDs were analysed using a beta-binomial GLMM, effects are expressed as changes in the odds of food remaining. Thus, odds ratios from the model represent the relative odds that food remained in the tray and are thus inversely related to foraging effort. For example, an odds ratio below 1 indicates that food was more likely to have been consumed (ie lower GUDs), reflecting greater foraging effort at the patch.

Results are reported using the language of evidence, with emphasis on the strength of support for observed effects rather than binary significance thresholds (as per Muff et al. 2022). This approach is applied consistently across both experiments.

### Field trapping experiment

#### Study location and details

To test whether phantom decoys can increase trap success, we conducted 2 large-scale trapping trials in crops around Walpeup, Victoria, Australia across 2 separate years (April 2022 and 2023, respectively). Winter crops are planted in April–May, with crops at our site generally rotating between wheat and canola. Crops are harvested in late spring to early summer. However, large quantities of spilt and unharvested grain remain on crops, providing an excess supply of background food for mice (Henry et al. 2022), which contributes to their low captures rates (Krebs et al. 1994).

#### Trapping protocols and treatments

Elliott traps were placed approximately 20 m apart and randomly allocated to a treatment in both years. We used 3 treatments: phantom decoy, decoy in trap, and wheat only. All traps were baited with a small handful of wheat, which is standard practice for mouse research in cereal crops (eg Ruscoe et al. 2023). Phantom decoys comprised a small ball of universal bait mix (∼28 g) in a cappuccino shaker (stainless-steel container with a mesh lid), which allowed mice to smell the bait without being able to access it. The phantom decoy treatment comprised an Elliott trap with a phantom decoy attached to a wooden stake fixed into the ground immediately outside the entrance of the trap (Figure S2). The decoy in trap treatment consisted of a decoy (steel shaker with universal bait ball) being placed inside the Elliott trap. This treatment was a procedural control for a potential attraction effect of the decoy itself. Universal bait mix is a highly preferred food source for mice (eg peanut butter, Witmer et al. 2014; and peanuts, Price and Banks 2017), and its odor presumably attracts more mice to traps, potentially leading to higher capture rates. Decoys placed inside traps would emit similarly attractive odors but would not function as phantom decoys because accessing them is part of the same decision to enter the trap.

#### Trapping design

In 2022, 120 Elliott traps were set in a large grid measuring approximately 200 m wide and 320 m long. An additional 30 traps were set in a second grid (100 m × 160 m) 3 nights later, bringing the total number of traps to 150. Thus, there were 40 traps per treatment for the first 3 nights, and 50 traps per treatment for nights 4 to 6. To assess whether trap visitation varied by treatment, we recorded mouse visitations at 45 traps (*n* = 15 per treatment) using infrared motion-triggered cameras (BolyGuard SG2060-X, settings: 30 s video, 0 s interval between triggers, high sensitivity, 1,280 × 720 video resolution) fastened to wooden stakes and positioned to film an area of approximately 40 cm in diameter around the entrance to the trap (Figure S2).

In 2023, waterlogged soil prevented harvesting of some crop sections and higher than average mouse numbers were reported in these areas. We established 4 grids of Elliott traps (28, 36, 36, and 20 traps) around these dense, unharvested sections, totaling 120 traps (*n* = 40 per treatment). Treatments were systematically allocated to traps to ensure the same number of traps per treatment per block.

All traps were set for a total of 6 nights (apart from 30 traps in 2022 which were set for 3 nights) and were checked each morning. Mice were shave marked according to the night they were caught, with mice caught multiple times given both marks to indicate that they had been recaptured. Fresh bait balls were added to phantom decoys both outside and inside traps after 3 nights.

#### Statistical analysis

We fitted a generalized linear mixed model (GLMM) with a binomial distribution and logit link function in R version 4.4.1 (R Core Team 2024) using the “lme4” package (Bates et al. 2015) to test whether capture and recapture probability varied according to treatment, night, and whether a capture had occurred in the trap on the previous night (previous night capture). Interactions between treatment, night, and previous night capture were also tested. Trap ID and grid were included as random effects to account for repeated measures and variation among trapping grids, respectively. However, grid was excluded from the final model based on model selection using Akaike's Information Criterion (AIC). We also conducted a post hoc power analysis using G*Power (v3.1.9.7) (Faul et al. 2009) to examine the relationship between sample size and detectable effect sizes in our logistic regression model. A significance level of 0.05% and 80% power (1 − *β*) were used. The baseline probability of the outcome and the observed odds ratio were derived from our fitted logistic regression model. Random effects were not included in the analysis.

We used bootstrapping with 10,000 replicates to estimate the mean capture rates and their 95% confidence intervals for each treatment (Phantom Decoy, Decoy in Trap, Wheat only) using the “boot” package in R (Canty and Ripley 2024). Pairwise differences between treatments were calculated for each bootstrap replicate, and 95% confidence intervals for these differences were constructed using bias-corrected and accelerated (BCa) intervals.

We fitted a negative binomial regression model using the “glmmTMB” package to test if visitations to traps with cameras in 2022 varied by treatment and night and included trap ID as a random effect.

## Results

### Foraging experiment

The effects of decoy presence and risk on GUDs were best described by a model including an interaction between decoy presence and risk, but not between decoy presence and food type. There was no evidence of a decoy × food interaction (*P* = 0.22) and model fit was not improved by including it (ΔAIC = 0.4), so the interaction was therefore excluded from the final model for parsimony.

**Figure 2:**
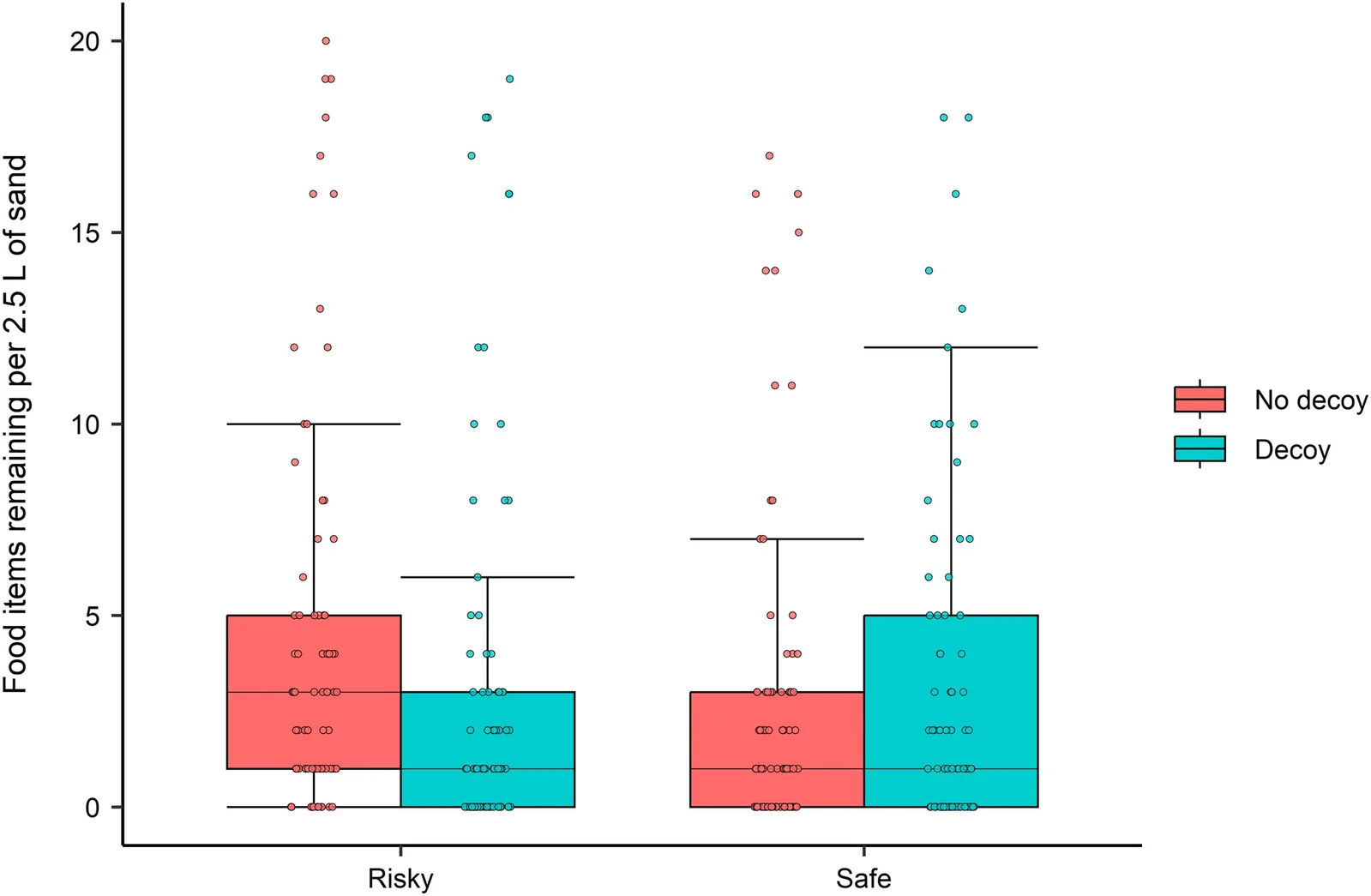
The number of food items remaining per 2.5 L of sand (ie giving-up densities; GUDs) in risky and safe foraging trays without (red) and with (blue) phantom decoys. Boxplots show median (center lines), interquartile range (IQR) (box limits), and data range (1.5 times the IQR) (whiskers). Individual data points are overlaid as jittered points to show the distribution and variability of GUDs within each group. Jittered points are offset slightly to avoid overlap and aid visualization of raw data.

Comparisons of estimated marginal means showed moderate evidence that mice left more food (ie higher GUDs) in risky than safe patches, but only in the absence of decoys, with the odds of food remaining 97% higher in risky patches when no decoys were present (Odds ratio (OR) = 1.97, 95% CI = [1.35, 2.89], *P* < 0.001). There was strong evidence that decoy presence increased foraging in risky patches, resulting in lower GUDs, with a 51% reduction in the odds of food remaining where decoys were present (OR = 0.49, 95% CI = [0.31, 0.79], *P* = 0.003). In contrast, there was no evidence that decoy presence altered the amount of food remaining in safe patches (OR = 0.97, 95% CI = [0.60, 1.57], *P* = 0.9) (Fig. 2).

There was strong evidence of an overall effect of food quality on GUDs, with the odds of food remaining in barley trays 2.51 times higher than in sunflower trays (OR = 2.51, 95% CI = [1.90, 3.33], *P* < 0.001). There was also strong evidence that GUDs decreased over time, with the odds of food remaining 35% lower on night 2 than on night 1 (OR = 0.65, 95% CI = [0.53, 0.81], *P* < 0.001).

There was no evidence for an interaction between decoy presence and food type. However, comparisons of estimated marginal means suggested that in risky areas, consumption of barley tended to be higher where decoys were present (*P* = 0.063), whereas there was no evidence of a decoy effect on barley GUDs in safe patches (*P* = 1.0).

**Figure 3:**
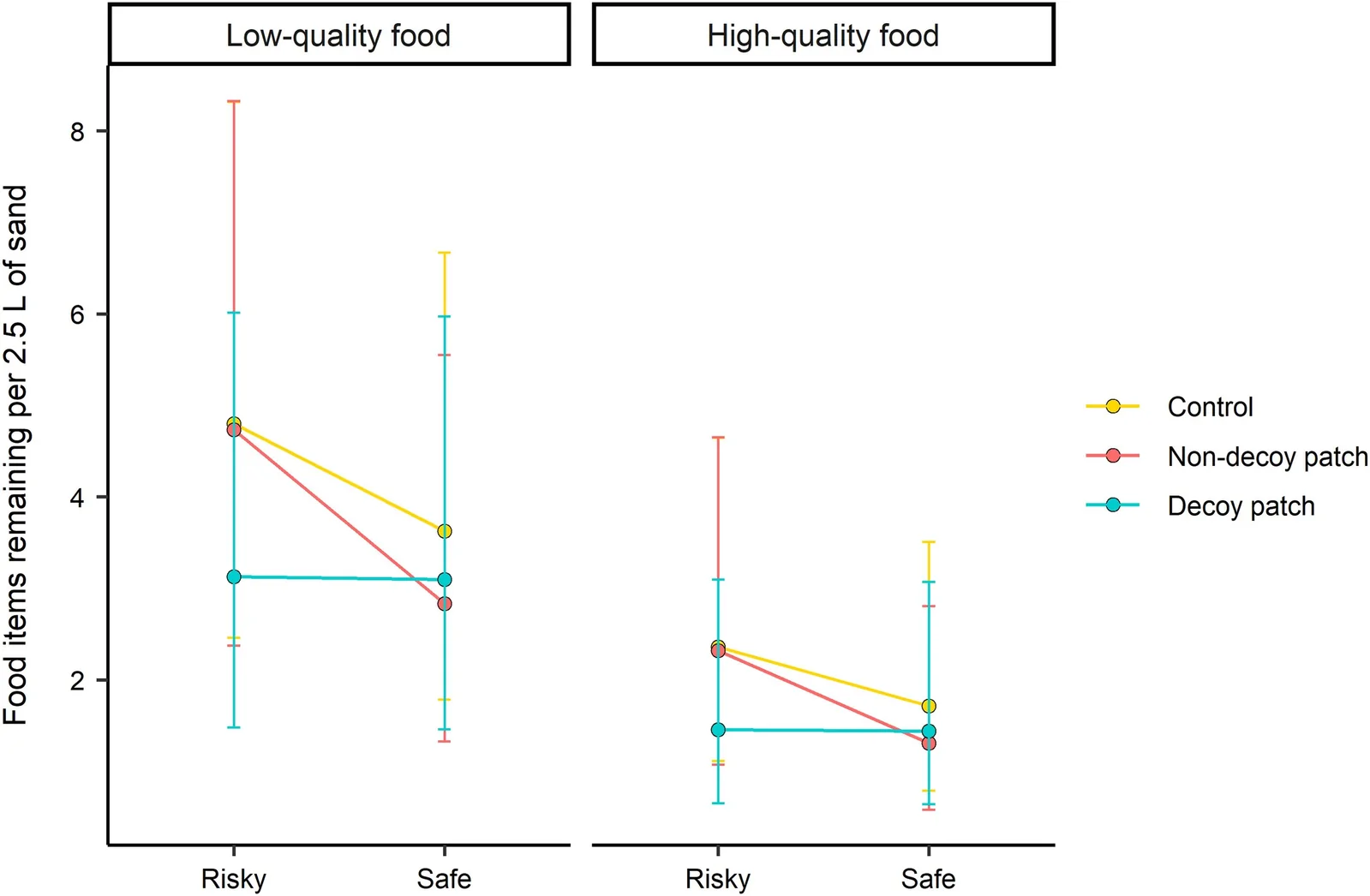
Predicted number of food items remaining per 2.5 L of sand (ie giving-up densities; GUDs) in risky and safe trays across control patches (gold), nondecoy patches (red), and decoy patches (blue), shown separately for low-quality food (barley) and high-quality food (sunflower). Points show estimated marginal means from the fitted beta-binomial model, averaged over night, and error bars indicate 95% confidence intervals. Predicted values are shown on the response scale and converted from proportions remaining to the number of food items remaining per tray, based on an initial 20 food items.

Analysis including enclosures both with and without phantom decoys showed weak evidence that responses to risk differed among patch types (control patches, nondecoy patches, decoy patches) (χ^2^(2) = 5.63, *P* = 0.060). Comparisons of estimated marginal means showed that less food was generally eaten (ie GUDs were higher) in risky than in safe trays at both control and nondecoy patches, indicating risk avoidance. At control patches (patches in enclosures without decoys), this pattern was weak for low-quality food (barley; OR = 1.28, 95% CI = [0.92, 1.78], *P* = 0.143) but stronger for high-quality food (sunflower; OR = 1.66, 95% CI = [1.13, 2.44], *P* = 0.009). At nondecoy patches (patches without decoys in enclosures where decoys were present), there was consistent evidence of risk avoidance for both low-quality (OR = 1.71, 95% CI = [1.08, 2.70], *P* = 0.025) and high-quality food (OR = 2.14, 95% CI = [1.28, 3.57], *P* = 0.006). In contrast, there was no evidence that risk influenced GUDs at decoy patches for either low-quality (OR = 0.94, 95% CI = [0.59, 1.49], *P* = 0.798) or high-quality food (OR = 1.11, 95% CI = [0.69, 1.80], *P* = 0.707), indicating similar foraging effort between risky and safe trays (Fig. 3).

### Field trapping experiment

**Figure 4:**
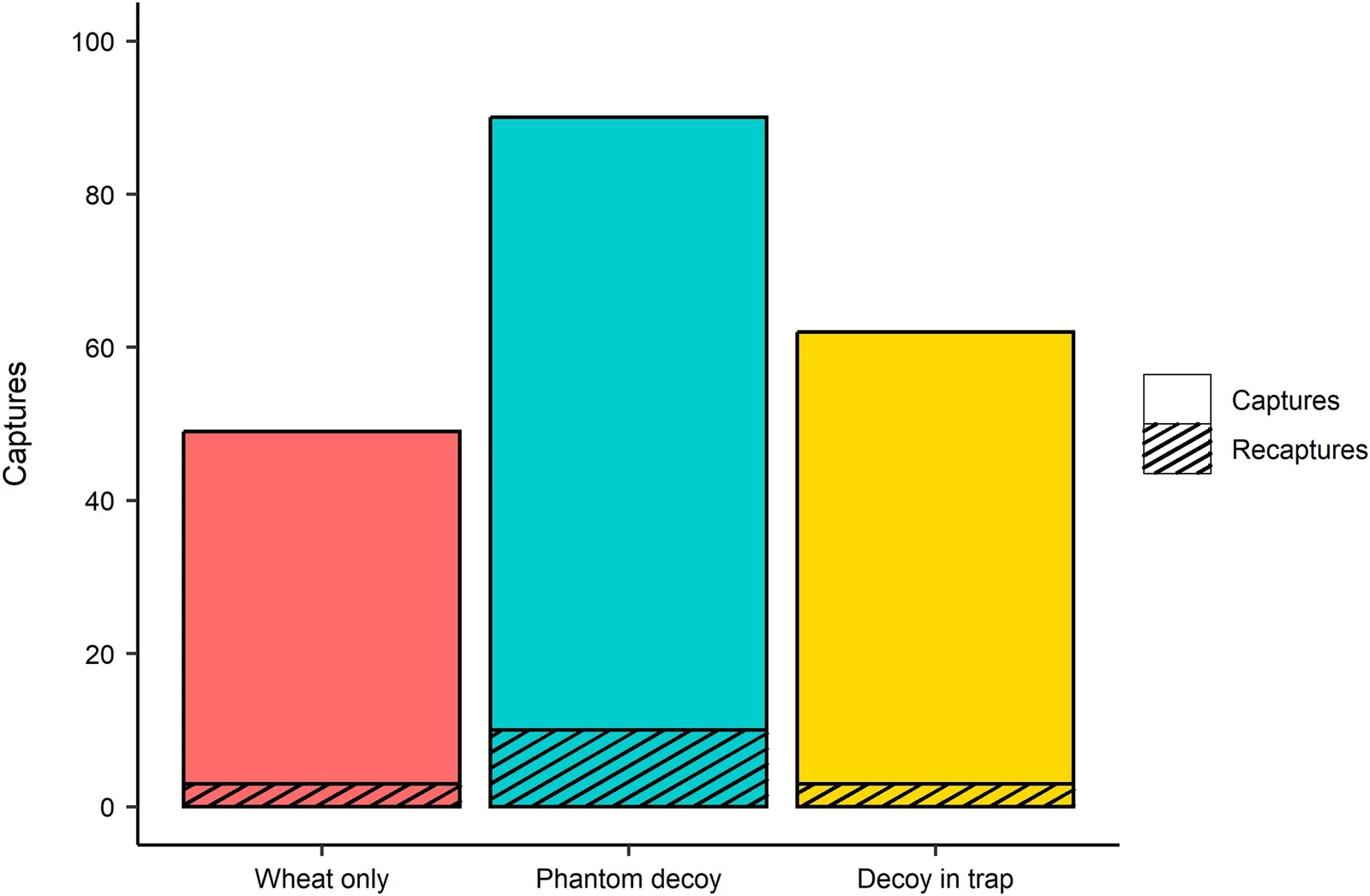
Total number of captures (color only) and recaptures (color + stripes) for each of the 3 treatments: wheat only (red; left; *N* = 91 traps; 516 nights), phantom decoy (blue; centre; *N* = 88 traps; 498 trap nights), decoy in trap (yellow; right; *N* = 91 traps; 516 trap nights).

Raw capture data showed that mouse captures and recaptures were highest in traps with phantom decoys (Fig. 4). However, there was no strong evidence that treatment influenced the odds of capture overall (χ^2^(2) = 4.17, *P* = 0.125). Post hoc pairwise comparisons using Tukey's adjustment provided only weak evidence that the odds of capture differed between phantom decoy and wheat only traps (*P* = 0.104) (Fig. 4).

Model estimates suggested that the odds of capture were 96% higher in phantom decoy traps than wheat-only traps (OR = 1.96, 95% CI = [1.03 to 3.75], *P* = 0.042), but there was no evidence that capture odds differed between decoy traps and procedural control traps. There was also no evidence that capture odds differed between procedural control traps and wheat only traps.

The odds of capture increased by approximately 12% each night (OR = 1.12, 95% CI = [1.0 to 1.26], *P* = 0.049), and capture odds were also 3.57 times higher in traps where captures had occurred on a previous night (χ^2^(1) = 17.7, *P* < 0.001; OR = 3.57, 95% CI = [1.99 to 6.40], *P* < 0.001). Random effects estimates indicated substantial variability among traps (*σ*^2^ = 1.911), suggesting that trap-level differences contributed meaningfully to variation in capture outcomes.

There was no evidence for a difference in the odds of capture between phantom decoy traps and procedural control traps. Post hoc power analysis indicated that a sample size of approximately 7,000 trap nights would be required to detect an OR of 1.43 (our observed effect size) with 80% power. Given our actual sample size of 1,530 trap nights, the smallest detectable OR with 80% power was 2.02.

**Figure 5:**
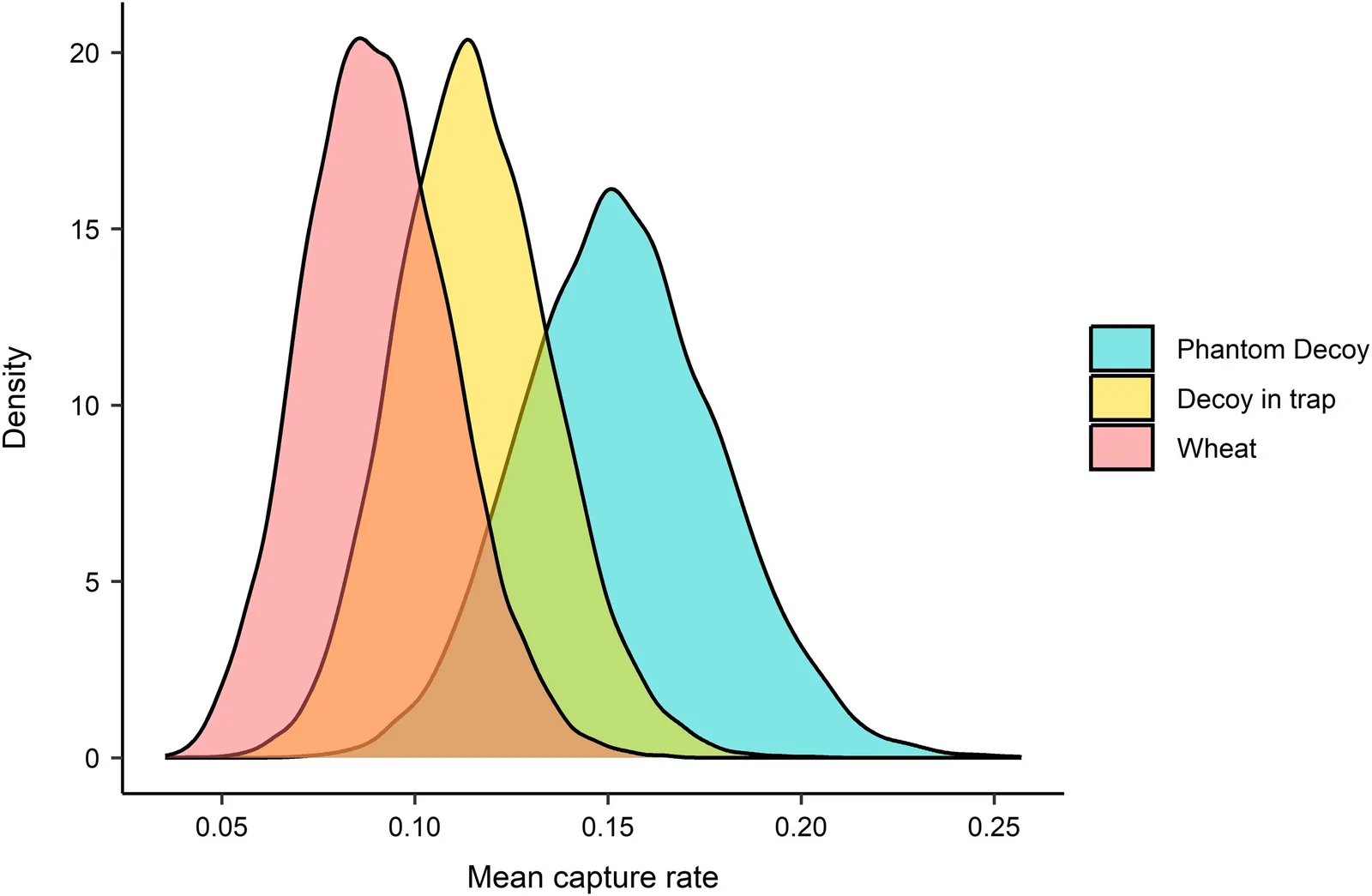
Kernel density plot of the mean capture rate for the 3 treatments: wheat only (red), decoy in trap (yellow), phantom decoy (blue). The density curves represent the estimated probability distribution of mean capture rates calculated across replicates for each treatment. Mean capture rate (proportion of successful captures) is on the *x*-axis. Probability density is on the *y*-axis, scaled such that the total area under the curve equals 1. Density curves were derived using bootstrapped mean capture rates, with 10,000 resamples.

Mean capture rates (total captures per trap divided by number of trap nights) estimated from bootstrapping revealed that capture rates for wheat only traps were 6% lower [95% CI = 0.003 to 0.1278] than those at phantom decoy traps. However, there was no evidence that mean capture rates for procedural controls (decoy inside trap) differed from those at phantom decoy traps (Fig. 5), although procedural control traps and wheat only traps did also not differ.

There was no evidence that treatment affected visitations to traps.

## Discussion

We found that phantom decoys changed the risk sensitivity of mice foraging in GUD trays and appeared to increase trap success, consistent with the hypothesis that decoys act to alter perceptions of MOCs. Mice left lower GUDs (thus spent more time foraging) at risky trays when a phantom decoy was present, despite no actual increase in patch value. Thus, the presence of a decoy alone was enough to change decisions by mice about where to forage and what level of risk they were willing to accept while foraging.

Although mice may have been initially attracted to patches with decoys, any attraction effect to unrewarding phantom decoy food would be short lived, as it could not be accessed and thus did not add direct value to the patch (Shettleworth 2009). Moreover, mice use the smell of buried seed to find their food, and the decoy odor was not present in the trays themselves. Importantly, decoys did not lead to a uniform increase in foraging across all treatments, but a reduction in sensitivity to predation risk. If decoys simply acted through attraction or by increasing patch value, we would expect lower GUDs across all trays in patches containing decoys, rather than specifically reducing the difference between risky and safe trays with decoys.

GUDs reflect the foraging decisions made as patches deplete, which reduces a forager's harvest rate (effort for return). We therefore argue that phantom decoys altered how mice evaluated the benefits of remaining in a patch relative to alternatives. Foraging decisions by animals are well known to be context-dependent, and perceptions of MOCs shape the context in which a decision is made. Thus, our findings suggest that information about an inaccessible foraging opportunity can increase foraging effort and acceptance of risky foraging opportunities at patches because it decreases the perceived MOCs of foraging in the patch.

As well as ruling out attraction, our design isolates MOC as the mechanism behind the observed effect. The GUD is the number of food items remaining in a patch left by the last forager at the end of the night, and reflects the energetic cost, predation risk and MOC of foraging in the patch (Brown 1988). Patches with and without decoys only differed by the presence of an inaccessible food as a missed opportunity which could not be depleted. There were no differences in the cost of foraging amongst patches (ie groups of 4 trays), as all patches contained food of the same quality and accessibility. There were also no differences in predation risk, as GUD trays were placed in the same proximity to vegetation cover across all patches (Fig. 1). That GUDs were no different between safe and risky trays when at patches with decoys shows that mice perceived MOC to be lower when decoys were present, making them forego more efficient foraging at other patches. Differences of a few seeds may not make a massive difference to the satiation of a mouse that eats up to 60 seeds per night. Yet, finding the last 10% of seeds in a foraging patch (1 to 2 food items here) can take more than 3 times as long as the entire first 50% (Price and Correll 2001), substantially increasing the risk a forager incurs. Mice were in enclosures for multiple days with access to the entire enclosure before decoys were added, so they presumably had information about the opportunities in the enclosure. In similar captive experiments, rodents are typically acclimatized to their surroundings within a few nights, after which foraging patterns remain consistent (Kotler and Brown 1990; Pastro and Banks 2006). Yet, phantom decoys seemed to override previously acquired information by altering perceptions of MOCs.

Of the large number of experiments that have used the GUD framework since its inception, few (around 15% in 2013) have considered the role of MOCs, and most of them only in passing (see meta-analysis by Bedoya-Perez et al. 2013). Fewer still have manipulated MOCs (but see Morgan et al. 1997; Shochat et al. 2004; Vickery et al. 2011), and in each case, MOCs were altered by the addition of alternative food. To our knowledge, no studies have used sensory information to target animal *perceptions* of MOCs directly. The majority of GUD experiments focus on predation risk and foraging costs, and MOC is generally assumed to have limited influence, or to be constant across patches (Brown 1988). Yet, predation risk and foraging costs should ultimately underpinned by MOCs, as they are assessed relative to available alternatives. Here, we show for the first time that sensory information can alter animal perceptions of MOCs to change foraging decisions as if MOCs were different among patches, even though they were not. Our results suggests not only that MOC is an undervalued driver of foraging decisions, but that animal perceptions of MOCs should be considered in studies on animal foraging decisions.

Mice changed their foraging behavior in response to a food item that was unavailable (ie an irrelevant alternative), a behavioral response that has been considered “irrational” in some literature (eg Bateson et al. 2002). Yet, seemingly irrational behavior can be rational and adaptive when considered in its evolved context (Fawcett et al. 2014). The best decisions should be made by animals with complete information about the options in their environment. But having complete information is virtually impossible (Janetos and Cole 1981), given that environments change constantly and gathering information is cognitively and energetically taxing (Marsh 2002). Instead, animals use heuristics, which are decision rules based on limited information that have evolved as effective tools for fast and efficient decision-making (Gigerenzer and Todd 1999). Heuristics can outperform more complex models that use more information (Gigerenzer 2008), and allow decision-makers to “offload” cognitively costly processing to the structure of the environment (Todd and Gigerenzer 2007; Risko and Gilbert 2016). However, outside of the conditions under which they evolved, heuristics can appear irrational and be maladaptive (Marsh 2002; Owen et al. 2017). For example, animals that use light to guide decisions about foraging, reproduction and communication display maladaptive behavior in response to artificial night lighting (Longcore and Rich 2004). It is therefore plausible that the behavioral responses to phantom decoys shown in our experiment reflect evolved, rational responses to perceived missed opportunities in the environment.

Foraging animals acquire information about spatial and temporal changes in resource availability as they move through their environment, using decision rules to guide their foraging decisions. Decision rules for resource acquisition can be effective when resources are not distributed randomly but are spatially positively autocorrelated (Fawcett et al. 2014), which means that resources in a given location tend to predict resources at nearby locations (Koenig 1999). Area-restricted search is one decision rule that foragers have evolved as a response to spatial autocorrelation (Fawcett et al. 2014), where encountering a resource triggers intensive search of a limited area around the resource (Dorfman et al. 2022). Even though they aren’t available, phantom decoys can change how decision-makers perceive information about foraging opportunities (Orlando et al. 2023). In a foraging context, it is possible that the presence of a highly attractive resource may alter how animals evaluate the relative benefits of remaining in a patch versus pursuing alternative opportunities elsewhere. For example, some plants produce flowers containing no nectar to conserve resources, but continue to attract pollinators because they signal the presence of other nectar-bearing flowers on the same plant (Bell 1986). Volatile organic compounds emitted from a mature fruit can signal to frugivores that a food resource is available, even if the fruit is out of reach (Rodríguez et al. 2013). Decoys appeared to lower rather than raise perceived MOCs, consistent with the prediction that phantom decoys signal to mice that the patches containing them were superior relative to alternatives.

Phantom decoy information is well known to affect decision-making in both human and nonhuman animals. There are many descriptions of how phantom decoys change decisions (eg relative advantage, Tversky and Simonson 1993; similarity-substitution, Pettibone and Wedell 2000; Pettibone and Wedell 2007; and the reactance effect, Brehm and Brehm 2013; Scarpi and Pizzi 2013), but mechanistic explanations are generally lacking in the literature. Our findings suggest that phantom decoys change decisions by altering the decision-maker's perception of MOCs. These insights may provide a useful framework for further investigation into the cognitive and behavioral mechanisms underlying decoy effects.

Although we predicted that decoys could alter responses to either predation risk or food quality, the effect we detected appeared primarily on the risk axis, with only weak evidence that mice consumed more barley (the low-quality food) in risky trays when a decoy was present. This is consistent with Brown's patch-quitting equation, H=C+P+MOC, which predicts that the food remaining in a tray (the GUD) will reflect a combination of the metabolic (C), predation risk (P), and MOC of foraging in the tray (Brown 1988). In safe trays with high-quality food (low C and low P), decoys were less effective because MOCs were already low, given that alternative trays were either riskier, contained lower-quality food, or both. In risky trays with low-quality food (high C and high P), decoys had the most substantial effect on MOCs because MOCs were highest at these trays. While decoys decreased perceptions of MOCs and increased foraging at patches generally, we suggest that they have the greatest potential to alter animal decision-making in contexts where foraging involves high perceived risk or low-quality resources (eg when MOCs are high).

In our field trapping experiment, traps with phantom decoys present at the entrance captured the highest numbers of mice, which is promising for their potential use to improve trap success in the future. There was weak evidence that the odds of capture between traps with decoys was higher than in wheat only traps, suggesting that decoys may decrease the MOCs associated with traps. However, there was also no evidence that the odds of capture at traps with decoys inside them was higher than those with decoys inside, so we could not rule out decoys solely acting an attractant to the trap location. Before animals can make a decision about whether to interact with a trapping device, they must first detect it, and highly attractive phantom decoys likely attract some mice to trap locations. That being said, we did not detect any evidence that visitations to traps in 2022 varied by treatment. Additionally, median home range sizes over 5 to 10 d for house mice inhabiting wheat crops have been estimated at 0.037 ha (370 m^2^) and 0.119 (1,190 m^2^) for breeding and nonbreeding seasons, respectively (Chambers et al. 2000). Given our trap spacing (∼20 m between traps), detection probability over 6 nights should be similarly high for all traps, regardless of treatment, which suggests that increased trap detection was not responsible for increased captures.

The most influential predictor of capture probability in our model was whether a capture occurred in the trap on the previous night, regardless of treatment. Additionally, our random effect, trap ID, indicated substantial variability in capture probability among traps with only a third (90) capturing any mice. This could reflect attraction to traps carrying the scent of previous captures (Rowe 1970). We did not clean traps between nights to replicate typical field practice, so residual social cues may have contributed to this variation. However, mice are highly patchily distributed in wheat fields due to their shared use of burrows (Chambers et al. 1996), which likely further amplified trap-level differences in capture rates. Despite this variability, phantom decoy traps had the highest proportion of captures overall. Post hoc power analysis indicated that approximately 4 times as many trap nights would be required to reliably detect a difference in the odds of capture between decoy traps and the procedural control at the observed effect size. The sample size used in our study could only have detected an effect size of roughly double the odds of capture. Given the findings of our foraging experiment, we argue that there is likely to be a real effect of phantom decoys on the decision by mice to enter traps, but a larger sample size is needed to overcome high variability and the limited statistical power of a binary outcome variable.

Our study adds to the growing body of literature that shows how the sensory information animals use to guide foraging and decision-making can be exploited to alter animal behavior (Price et al. 2022). Animals sample and collect information about their environment as they forage in order to minimize MOCs, yet little attention has been given to how sensory information can be used to alter animal perceptions of MOCs and alter their decision-making. We provide the first evidence that animal perceptions of MOCs can be altered solely by the presence of an attractive but inaccessible resource (a phantom decoy), even though actual MOCs remained unchanged. The missed opportunity that we presented to mice changed their decisions about where they foraged, the quality of food they foraged for, and the risk they were willing to incur while doing so. Fundamentally, animal decisions will be motivated to minimize lost opportunities, and we suggest that responses to phantom decoys are evolved responses to information about foraging opportunities in the environment. Although phantom decoys did not increase the probability of capture compared with our procedural controls, they did alter perceptions of MOCs, highlighting their potential to increase interaction rates with devices such as traps. Our findings point to new avenues of research for improving management by incorporating relatively simple manipulations of sensory information, including phantom decoys. Understanding how such novel, yet simple approaches work should be the focus of future research.

## Supplementary Material

arag078_Supplementary_Data

## Data Availability

Analyses reported in this article can be reproduced using the data provided by Parker (2026).
