# Supplementary material for: Phantom decoys alter perceived missed opportunity costs and increase risky foraging decisions
Source: Behav Ecol. 2026 Jul 11;37(5):arag078. doi: 10.1093/beheco/arag078 (PMC13448324; doi:10.1093/beheco/arag078)
Supplement: Supplementary file 1 [file arag078_supplementary_data.docx]

**Supplementary material** – Phantom decoys alter perceived missed opportunity costs and increase risky foraging decisions

Supplementary figure 1

Figure 1 Aerial image of the nine mouse- and predator-proof enclosures used in our study. Inner perimeter fences measure 15 × 15 m and chicken wire fencing surrounds each of the enclosures.

Supplementary figure 2

Figure 2 Setup of a phantom decoy trap for the field trapping experiment. Elliott traps were placed parallel to rows of wheat stubble. A cappuccino shaker containing a 28-gram ball of universal small mammal bait mix (peanut butter, oats, honey) is attached to a wooden stake with duct tape and fixed in the ground. Cameras were positioned on 45 traps (n = 15 per treatment) in 2022 to record visitations by mice.
